# The Hospital World

**Published:** 1911-04-22

**Authors:** 


### The Hospital World.

### PERSONALIA.

#### Col. Warburton's Work at Edinburgh Royal Infirmary.

Last week reference was made to the appointment of Lieut.-Colonel Sir Joseph Fayrer, Bart., R.A.M.C., as Superintendent of the Edinburgh Royal Infirmary, and we are now in a position to add to that a few words concerning the progress of the institution under his predecessor. Col. Warburton has held the post of Superintendent for the past twelve years, and during that time many of the departments of the Infirmary have been renovated and brought up-to-date. A new out-patient department has been built and equipped, and, like all modern teaching institutions, a clinical laboratory has been added. Not the least of the reforms carried out under his administration is that of granting pensions to the sisters. During the past year the question of remuneration of the sisters was again the subject of inquiry by the managers, and the pension scheme which was adopted in 1901, providing for an annuity of £25 per annum for each sister who paid one-fourth share of the premium, was discarded, and the arrangement now come to is that the pension shall be £40 and that no payment on account of premium shall be required from the sisters. This pension begins at the age of fifty-five. Under Colonel Warburton's guidance the Edinburgh Royal Infirmary has gone steadily, if quietly, forward, and his reward lies in its efficiency to-day. His retirement, which led to thirty-two applications for the post—the list from which the final choice was made comprised Major C. C. Fleming, R.A.M.C., Inspector-General T. D. Gimlette, C.B., Haslar, Lieut.-Col. W. E. Jennings, I.M.S., Col. A. Peterkin, A.M.S., Lieut.-Col. A. F. Russell, R.A.M.C., and of course Sir Joseph Fayrer—was announced in The Hospital of December 3, 1910 (p. 303).

